# Study-Related Predictors for Depression, Suicidal Ideation and Suicide Risk in German Veterinary Medical Students

**DOI:** 10.3390/healthcare13080938

**Published:** 2025-04-19

**Authors:** Nadine Schunter, Mahtab Bahramsoltani, Luise Böhler, Heide Glaesmer

**Affiliations:** 1 Institute of Veterinary Anatomy, School of Veterinary Medicine, Freie Universität Berlin, 14195 Berlin, Germany; mahtab.bahramsoltani@fu-berlin.de; 2 Department of Medical Psychology and Medical Sociology, University of Leipzig, 04103 Leipzig, Germany; luise.boehler@medizin.uni-leipzig.de (L.B.); heide.glaesmer@medizin.uni-leipzig.de (H.G.)

**Keywords:** depression, suicidal ideation, suicide, effort, reward, effort–reward imbalance, overcommitment, ERI, time of study choice, grade, learning group, peer competition, performance expectation, study requirements, examination requirements, veterinary students, Germany

## Abstract

**Background/Objectives**: Compared to the general population in Germany, higher risks for depression, suicidal ideation and suicide risk have been reported for German veterinary students. This study assessed various demographic and study-related risk factors for depression, suicidal ideation and suicide risk for the first time. **Methods**: An online survey was conducted among German veterinary students to determine demographic and study-related characteristics, study conditions, depression, suicidal ideation, suicide risk, Effort–Reward Imbalance (ERI student version) and motivation and achievement goals (Achievement Goal Inventory) during studies. Data of 913 German veterinary students (90.7% female) aged 18 to 46 years (mean age 23.6 years) were analyzed (response rate 14.3%). Hierarchical logistic regression analyses were conducted, with depression, suicidal ideation and suicide risk as dependent variables and demographic as well as study-related factors as independent variables. **Results**: With the factors used, the variance explanation was highest for depression, followed by suicidal ideation and suicide risk. Low reward and high overcommitment were found to be the most important predictors for depression, suicidal ideation and suicide risk. Significant associations with depression, suicidal ideation and suicide risk were also revealed for time of study choice, general university entrance qualification grade, learning in a learning group, perceived peer competition and performance expectation from lecturers, as well as transparency of study requirements and transparency of examination requirements. **Conclusions**: The results of this study provide indications for the development of suitable prevention and intervention measures at veterinary medical schools to strengthen the mental health of veterinary students in Germany.

## 1. Introduction

The results of various studies from different countries indicate a significantly increased risk of depression, suicidal ideation and death by suicide among veterinarians [1,2,3,4,5,6]. According to several international studies, the risk of veterinarians dying by suicide is four times higher than that of the general population [1,2,3,4,5] and twice as high than among other health professionals [1]. The first study among veterinarians in Germany showed depression rates three times higher, suicidal ideation rates two times higher and suicide risk more than six times higher compared to the German general population [6].

For veterinary students, various studies revealed similar results. A cross-sectional investigation as well as a longitudinal investigation from the early 2000s already demonstrated that one-third of the first-year veterinary students surveyed showed symptoms of depression [7,8]. The results of a recent cross-sectional investigation among Swiss veterinary students indicated that veterinary students in all years of study showed higher levels of depression compared to the general population [9]. Furthermore, another very recent study among Swiss veterinary students reported that more than 50% of the students were screened positive for depression [10]. Several studies among US veterinary students found that one-third to two-thirds of students exhibit depressive symptoms, which indicates that compared with the US general population as well as with medical students, the prevalence of depression is higher among veterinary students [7,11,12,13,14,15]. Additionally, one of these studies indicated that almost one-third of the surveyed veterinary students reported having seriously thought about suicide, and almost 5% had made an attempt [14]. Also among UK veterinary undergraduates, evidence was found that they experience significantly higher rates of depression and suicidal ideation compared to the general population [16]. In the first study on depression, suicidal ideation and suicide risk among veterinary students in Germany, depressive symptoms were found in 49.9%, suicidal ideation in 19.9% and an increased suicide risk assessed with the Suicide Behaviors Questionnaire (SBQ-R) in 24.0% of the students. Compared with the German general population of the same age range, the veterinary students thus showed a twenty-twofold-higher risk of depression, a fourfold-higher risk of suicidal ideation and a fourfold-higher suicide risk [17].

Various studies have addressed specific risk factors in veterinarians, including the characteristics of individuals entering the profession, negative effects during undergraduate training, professional and social isolation, stigma associated with mental illness [1,18], job-related factors such as expectations of clients, challenging or demanding interactions with clients, long working hours including nights and weekends, overwork, insufficient control over workload, work–life imbalance [1,5,19], availability and familiarity with lethal means [1,19,20,21,22] and performing animal euthanasia [19,23,24]. Although the risk factors mentioned for veterinarians, such as performing euthanasia on animals and access to lethal means, as well as challenging job demands and interactions with clients, are primarily specific to veterinarians and cannot be directly transferred to veterinary students [19], some of the stressors experienced are similar to those that affect students [25]. The undergraduate veterinary program is intensive and demanding, with a time-consuming workload and continuous examination load [7,8,11,12,13,14,16,25,26,27,28,29,30,31]. Apart from these curricular conditions, further aspects were discussed as common risk factors, including chronic sleep difficulties, lack of time for social and recreational activities, family and personal relationship concerns, perception of difficulty fitting in with other students, competition between peers, unclear instructor expectations, feeling behind in studies, concerns about falling short in terms of academic outcomes and a hidden fear of failing [7,8,12,25,27]. These risk factors are similar to those reported by medical students [30], with the medical school curriculum and environment identified as the students’ principal sources of distress [13,31]. Furthermore, high achievement goals, like learning and performance motivation, are associated with depressive symptoms in adolescents [32]. In order to have more time to study, many veterinary students begin to sacrifice private areas of their lives [13]. Chronicity of this behavior during the entire study program can contribute to a study–life imbalance, emotional and social isolation, and stress, anxiety and depression [13,26,27]. In turn, this can lead to a reduced feeling of belonging to both the student and professional community [28].

Psychological distress can have negative effects on the capacity for learning and academic success [12,13]. In the period from 2008 to 2012, the number of UK university students seeking counseling rose by more than one-third [33]. Numbers have more than doubled at five universities in the UK and for veterinary students, an increase of up to 26.6% has been reported [34]. Results of a long-term study of counseling needs among Turkish university students indicated a decrease in mental health after the start of their studies [35,36]. Accordingly, the highest percentage of students who consulted the counseling service in recent years were female and suffered from mental health problems. Adjustment to university life, academic stressors and students’ concerns about their career opportunities were mentioned as possible causes [35,36]. According to Bartram and Baldwin, every period of the veterinary career, from the beginning with the characteristics of applicants to the veterinary program to retirement, can be examined to identify early risk factors and later triggers for suicidal behavior [1].

Therefore, this study aimed to explore the association of potential study-related risk factors with depression, suicidal ideation and suicide risk in German veterinary students.

## 2. Participants, Materials and Methods

### 2.1. Participants

The undergraduate veterinary program in Germany is a state examination program regulated by the German Veterinary Medical Licensure Law (TAppV) and is offered at five German veterinary schools. All German veterinary students enrolled in the veterinary program (N = 6367) in winter term 2018/2019 at all German veterinary schools were invited by email to answer an anonymous online questionnaire via Unizensus (Blubbsoft GmbH) from 15 November 2018 to 31 March 2019. The invitation included information about the study and its objectives as well as the link to the questionnaire and was distributed through the respective Dean’s Offices and Study Offices between 15 November and 17 December 2018. Furthermore, the veterinary student council initiatives of the five German veterinary schools, which are elected by the students and represent the student body, were asked to spread the call for participation among the students. Study Offices and veterinary student council initiatives generally hold the current email addresses of their respective students. A reminder was sent via email in March 2019 [17].

At the beginning of the survey, participants received detailed information about the research aim, potential advantages and risks of participation and data protection. Subsequently, the study participants provided their informed consent by clicking on ‘agreement’ to be given access to the questionnaire [17].

Prerequisites for participation were enrollment as a veterinary student in one of the five German veterinary schools as well as adequate German language skills. In total, 922 veterinary students answered the questionnaire; 9 participants were excluded from the analysis due to more than 25% missing data per scale or unreliable information about depressive symptoms, suicidal ideation and suicide risk. Consequently, 913 German veterinary students (90.7% female, n = 828) were subject to the analyses presented here. Thus, the sample represented 14.3% of the 6367 veterinary students enrolled at the five German veterinary schools in the winter term of 2018/2019 [17,37]. Of these, 37 respondents did not provide any information about their age and 1 participant indicated diverse gender. The latter participant was not assigned to the male/female group analyses or the regression analyses.

This study was reviewed and approved by the Ethics Committee of the Medical Faculty of the University of Leipzig (No. 139/18-ek).

### 2.2. Demographic Data

The survey started by collecting some demographic data. Participants indicated their age in years, as well as their gender as female, male or diverse. They were asked when they made the decision to study veterinary medicine (‘time of study choice’: before primary school, during primary school, during lower secondary level, during upper secondary level, at the time of upper secondary school leaving examination, during an orientation period after leaving secondary school, or during vocational training/during another study program). The students were requested to provide their general university entrance qualification grade (‘grade’), whether they had completed a study-relevant vocational training (‘vocational training’) and the semester in which they were currently enrolled in veterinary medicine. Participants also indicated their main source of financing for their studies. Possible answers were as follows: own income from gainful employment, support from parents and/or other relatives, support from their (spouse) partner, funding under the Federal Training Assistance Act (BAföG), loan (e.g., education loan/loan to finance studies) and scholarship. For the analysis, the response options were grouped into own income from gainful employment (‘employment income’) and other financial sources (‘other financing’). The latter includes all answer options except ‘employment income’.

### 2.3. Study Conditions

Based on several potential risk factors that have already been mentioned in the literature, 13 items forming three scales were designed to assess the study conditions. For this purpose, psychometrically validated items were selected from the questionnaire for the evaluation of the bachelor’s programs at Freie Universität Berlin. Some of them were adapted in their wording to reflect the conditions and structure of the veterinary program more accurately [38,39,40,41,42]. Clarity and transparency of study and examination requirements consist of the two items “study requirements are clear and transparent” (‘transparency of study requirements’) and “examination requirements are clear and transparent” (‘transparency of examination requirements’) [7,8,25,27,28,30,41,42]. For assessing the social climate between students as well as between students and lecturers, the following 5 items were used: “I feel I am in strong competition with my peers regarding academic performance” (‘peer competition’) [7,8,11,25,28,29,30,41], “I feel that the working atmosphere among the students is pleasant” (‘students’ working atmosphere’) [7,8,12,29,41,42], “I often learn with other students in a learning group” (‘learning group’) [7,8,12,25,30,41,42], “I feel a strong performance expectation from the lecturers” (‘performance expectation from lecturers’) [25,27,30,31,41] and “I feel that the working atmosphere with the lecturers is pleasant” (‘working atmosphere with lecturers’) [12,25,28,30,41]. Items of the ‘transparency of study requirements’ and ‘social climate’ scales were scored on a 6-point Likert scale from 1 (‘strongly disagree’) to 6 (‘strongly agree’). In addition, students assessed their satisfaction with the support and supervision provided by the lecturers by rating the following 6 items: “teaching of learning techniques and strategies” (‘teaching learning strategies’) [12,25,30,41,42], “support with learning and work difficulties” (‘support with learning difficulties’) [12,41,42], “support in performing practical skills” (‘support in practical skills’) [12,28,41,42], “support in preparing for examinations” (‘support in exam preparations’) [12,30,41,42], “feedback on individual academic and examination performance” (‘receiving feedback’) [12,30,31,41,42] and “contactibility/availability for questions about the study program” (‘lecturers’ contactibility’) [12,25,30,41,42]. A 6-point Likert scale was used for the latter items from 1 (‘extremely dissatisfied’) to 6 (‘extremely satisfied’).

### 2.4. Instruments

#### 2.4.1. PHQ-9

To assess depressive symptoms over the last two weeks, the depression module of the Patient Health Questionnaire (PHQ-9) was used [43]; German version [44]. The PHQ-9 is a self-administered questionnaire with 9 items that evaluates the following criteria according to the Diagnostic and Statistical Manual of Mental Disorders, 4th Edition (DSM-IV): depressed mood; markedly diminished interest or pleasure in most or all activities; significant weight loss (or poor appetite) or weight gain; insomnia or hypersomnia; psychomotor retardation; fatigue or loss of energy; feelings of worthlessness or excessive or inappropriate guilt; diminished ability to think or concentrate, or indecisiveness; and recurrent thoughts of death (not just fear of dying), or suicidal ideation [45]. The total score ranges from 0 to 27, since each of the items on a 4-point Likert scale can be scored from 0 (‘not at all’) to 3 (‘nearly every day’) [43,46]. Scores are divided in categories suggesting varying levels of depression: 0–4 = none to minimal, 5–9 = mild, 10–14 = moderate, 15–19 = moderately severe and 20–27 = severe depression. A total score of 10 or above indicates at least clinically relevant symptoms of depression [43,46]. The German version of the PHQ-9 shows excellent criterion validity compared to a clinical interview, with a sensitivity of 95% and a specificity of 86% as well as good internal consistency (Cronbach’s α = 0.88) [46].

For assessing suicidal ideation, item 9 of the PHQ-9 (‘Thoughts that you would be better off dead, or of hurting yourself in some way’) was used. Participants with a score from 1 (several days) to 3 (nearly every day) are categorized as being screened positive for current suicidal ideation [43,46].

#### 2.4.2. SBQ-R

The Suicide Behaviors Questionnaire-Revised (SBQ-R), a 4-item self-report measure recommended for population-based research [47] and validated for use in non-clinical adolescent and adult participants [48], has been applied to reliably and validly assess different aspects of suicidality [48]; German version [49]. In detail, it comprises lifetime suicidal ideation and suicide attempt; frequency of suicidal ideation over the past 12 months; threat of suicidal behavior; and likelihood of death by suicide in the future [48]. The total score ranges between 3 and 18. An established cut-off-score of 7 or higher is usually used to select participants with an increased suicide risk [48]. The internal consistency of the SBQ-R is satisfactory (Cronbach’s α = 0.72) [49].

#### 2.4.3. Effort–Reward Imbalance Questionnaire

The original effort–reward imbalance (ERI) model focuses on work-related reciprocity between effort invested and reward received. It postulates that the quantified imbalance as a ratio term between high effort spent and low reward received in turn causes strong negative emotions and sustained stress reactions [50,51,52,53,54]. Overcommitment is described as a specific personal coping strategy [52] that can intensify the effects of effort–reward imbalance on health [51,52,55]. Therefore, the strongest effects are expected in people with high scores on overcommitment [54]. The ERI model was extended to the context of university and an adapted short student ERI questionnaire was developed [52,53,54]. According to this, perceived university distress among students also results from the imbalance between effort, such as a heavy study load, and low reward, such as a missing respect from supervisors [53,56]. Among university students, an imbalance between effort and reward as well as a high overcommitment may also represent mental health determinants [54,55,57]. To assess effort–reward imbalance in university settings, the German short student version of the ERI questionnaire, showing satisfactory psychometric properties (Cronbach’s α for effort = 0.67, reward = 0.65 and overcommitment = 0.79), was used [52]. This self-administrated questionnaire consists of three subscales, with 14 items in total that represent all dimensions of the original effort–reward imbalance model: ‘effort’ (extrinsic component, 3 items), ‘reward’ (extrinsic component, 6 items) and ‘overcommitment’ (intrinsic component, 5 items). All items use a 4-point Likert scale from 1 (‘strongly disagree’) to 4 (‘strongly agree’) [51,52,58,59,60,61]. For each scale, the total value is calculated. For the scale ‘effort’, this can range between 3 (low) and 12 (high), for the scale ‘reward’ between 6 (low) and 24 (high) and for the scale ‘overcommitment’ between 5 (low) and 20 (high). Since there are no defined cut-off scores for the subscales, the higher the scores for a scale, the more effort, reward or overcommitment is indicated [61]. In addition, the effort–reward ratio (ER ratio) was calculated using the following formula: c E/R, where “c” is a correction factor for unequal numbers of items in the nominator and denominator (c = 6/3), “E” is the total score of the effort scale and “R” is the total score of the reward scale [61]. Thereby, a ratio greater than 1 indicates an imbalance between high effort spent and low reward received [51,61,62].

#### 2.4.4. Achievement Goal Inventory

To investigate the main motivation and achievement goals during studies, the German version of the Achievement Goal Inventory was used in the four-scale version, which showed very good psychometric properties [63]; German version [64]. The following items were applied to assess whether students learn for the purpose of learning or mastering challenges (‘learning/challenge goals’: “I strive to constantly learn and improve in my courses”, “In school I am always seeking opportunities to develop new skills and acquire new knowledge”, “In my classes I focus on developing my abilities and acquiring new ones”, “I really enjoy facing challenges, and I seek out opportunities to do so in my courses” and “It is very important to me to feel that my coursework offers me real challenges”), of improving their skills (‘ability goals’: “It is important to me to confirm my intelligence through my schoolwork”, “In school I am focused on demonstrating my intellectual ability” and “One of my important goals is to validate my intelligence through my schoolwork”), of achieving good grades (‘outcome goals‘: ”I really want to get good grades in my classes” and “A major goal I have in my courses is to perform really well”), or because they want to be better than their peers or achieve better grades (‘normative goals’: “I try to do better in my classes than other students”, “A major goal I have in my courses is to get higher grades than the other students” and “When I take a course in school, it is very important for me to validate that I am smarter than other students”). Items on a 7-point Likert scale can be scored from 1 (‘strongly disagree’) to 7 (‘strongly agree’). The mean value for each scale ranges from 1 to 7. The higher the mean value, the stronger the respective goal orientation [63,64].

### 2.5. Statistical Analyses

Statistical analyses were conducted with IBM SPSS 24.0 for Windows.

Descriptive statistics were used for the description of the demographic and study-related details. As with Schunter et al. (2022), a tolerance criterion of 25% missing values per scale was employed [17]. If participants answered less than 75% of the items, the data of the respective scale were excluded. By using person mean imputation, the missing data were substituted by the individual mean score [17].

Hierarchical logistic regression analyses were used to test the association of demographic factors, ‘effort’, ‘reward’ and ‘overcommitment’ in university settings (student ERI), motivation and achievement goals during studies (Achievement Goal Inventory) and study conditions (clarity and transparency of study and examination requirements, social climate, support and supervision provided by the lecturers) with depression, suicidal ideation and suicide risk. Therefore, 6 different models were compared. Model 1 comprises gender, age, ‘time of study choice’, ‘semester’, ‘grade’, ‘vocational training’ and ‘employment income’. For the other models, the following aspects were added to those included in model 1: ‘transparency of study and examination requirements’ (model 2), the values regarding social climate (model 3), the aspects of support and supervision provided by the lecturers (model 4), the three scales of the student ERI (model 5) and the four scales of the German version of the Achievement Goal Inventory (model 6). For reporting the estimates, odds ratios (ORs) with 95% confidence intervals (CIs) were used. Values were considered significant at *p* < 0.05. For assessing the amount of variance accounted for the predictor variables, adjusted R^2^ values are presented.

## 3. Results

### 3.1. Demographics

The average age of the German veterinary students (N = 913, 90.7% female, n = 828 vs. 9.2% male, n = 84) was 23.6 years, ranging from 18 to 46 years. Compared with the gender distribution of the entire population of German veterinary students for the winter term 2018/2019 (85.4% female, n = 5440 vs. 14.6% male, n = 927) [37], the proportion of women in this study was approximately 5% higher and of men approximately 5% lower. Therefore, generalizing the results for men to the entire population of German veterinary medical students is only possible to a limited extent. The mean of the grade was 1.84. Within the German grading system, which ranges from 1.0 (excellent) to 6.0 (insufficient), this mean value reflects very good performances in a German upper secondary school.

Students from all five years of the study program participated in the survey, with students in the third and fifth semesters being most frequently represented, followed by the students from the seventh, eleventh, first and ninth semesters. With 16.9% (n = 154), the majority of students decided to study veterinary medicine during primary school years. Respectively, about 15% of the responses are evenly distributed between the time during upper secondary level, during an orientation period after leaving secondary school and during vocational training or during another study program. Every fifth participant (22.1%, n = 201) reported having a ‘vocational training’. A small proportion of veterinary students (8.9%, n = 81) stated that they earn ‘employment income’ (Table 1).

### 3.2. Study-Related Factors

The following study-related items were scored on a 6-point Likert scale, with scores between 1 and 2 rated as low, 3 and 4 as moderate and 5 and 6 as high. As displayed in Table 2, participants rated the ‘transparency of study requirements’ with a total mean value of 3.91 (SD = 1.21) and of ‘examination requirements’ with 3.60 (SD = 1.27). The total mean scores for the social climate items were 2.96 (SD = 1.47) for ‘peer competition’, 4.23 (SD = 1.26) for the ‘students’ working atmosphere’, 3.21 (SD = 1.65) for ‘learning group’, 4.72 (SD = 1.19) for ‘performance expectation from lecturers’ and 3.90 (SD = 1.09) for ‘working atmosphere with lecturers’.

In terms of satisfaction with the support and supervision provided by the lecturers, the total mean score for ‘teaching learning strategies’ was 2.89 (SD = 1.39), for ‘support with learning difficulties’ 2.58 (SD = 1.23), for ‘support in practical skills’ 3.47 (SD = 1.37), for ‘support in exam preparations’ 2.78 (SD = 1.23), for ‘receiving feedback’ 2.69 (SD = 1.31) and for satisfaction with ‘lecturers’ contactibility’ 3.79 (SD = 1.44).

The total mean value of the ‘effort’ scale was 9.26 (SD = 1.60), that of the ‘reward’ scale 15.54 (SD = 3.11) and of the ‘overcommitment’ scale 14.25 (SD = 3.67). The calculated effort–reward ratio was 1.19 and thus higher than 1.0. Therefore, an imbalance between high effort spent and low reward received was indicated. However, when taking gender into account, it becomes apparent that the imbalance between effort and reward more often affects female students (effort–reward ratio: 1.21), while male students have an almost balanced ratio between effort and reward on average (effort–reward ratio: 1.06).

With regard to motivation and achievement goals during studies, participants scored the goal of achieving good grades (‘outcome goals’) with a total mean of 4.66 (SD = 1.51), the improvement of skills (‘ability goals’) with 3.95 (SD = 1.49), the goal of learning and mastering challenges (‘learning/challenge goals’) with 4.81 (SD = 1.04) and ‘normative goals’ (being better than peers and achieving better grades) with 2.32 (SD = 1.34).

### 3.3. Potential Risk Factors Associated with Depression

Including the demographic variables (model 1), age, ‘time of study choice’ and ‘grade’ were found to be significant predictors for depression. Later, ‘time of study choice’ was associated with decreased risk of depression, while a better ‘grade’ was related to an increased probability of depression.

When ‘transparency of study and examination requirements’ was added (model 2), age was no longer a significant predictor. Transparent examination requirements were associated with decreased risk of depression.

By adding social climate variables in model 3, age and ‘transparency of examination requirements’ were no longer significant. ‘Learning group’ was associated with a lower risk of depression, while the perception of high ‘peer competition’ was significantly and positively associated with depression. Additionally, a higher feeling of ‘performance expectation from lecturers’ was related to an increased likelihood of depression.

After adding satisfaction with support and supervision provided by lecturers (model 4), nothing fundamentally changed for demographic, ‘transparency of study and examination requirements’ and social climate variables. The only significant association found was with ‘support in exam preparations’ decreasing the likelihood of depression.

By including the three ERI subscales in model 5, all variables except ‘learning group’ no longer had significant association with risk of depression. All ERI variables had a highly significant association, with low ‘reward’, high ‘effort’ and high ‘overcommitment’ increasing the likelihood of depression.

Adding the Achievement Goal Inventory scales in the final step (model 6), ‘time of study choice’ again became significant, and the results for the ERI variables remained unchanged. The Achievement Goal scale ‘ability goals’ was significantly and positively associated with the probability of depression.

The highest proportion of variance was explained by model 5, followed by model 3, model 1 and model 2, while model 4 and model 6 were almost negligible in terms of variance explanation (Table 3).

### 3.4. Potential Risk Factors Associated with Suicidal Ideation

Regarding the demographic variables in model 1, suicidal ideation was significantly associated with ‘grade’, with a better ‘grade’ being associated with an increased likelihood of current suicidal ideation.

After including the variables of ‘transparency of study and examination requirements’ in model 2, the ‘grade’ variable remained unchanged. Additionally, study requirements were significantly negatively associated with suicidal ideation.

After including the social climate variables in model 3, the ‘grade’ variable remained nearly unchanged. ‘Transparency of examination requirements’ as well as ‘performance expectation from lecturers’ were significantly and positively associated with suicidal ideation, while ‘learning group’ and ‘working atmosphere with lecturers’ were significantly and negatively associated with suicidal ideation.

By adding the variables satisfaction with the support and supervision by the lecturer in model 4, the significant association of suicidal ideation with ‘grade’, ‘transparency of examination requirements’ and ‘learning group’ remained unchanged, while the association with the ‘working atmosphere with lecturers’ was no longer a significant predictor. In addition, this model revealed a significantly negative association of ‘support in exam preparations’ with suicidal ideation.

When including the three ERI scales in model 5, the variables ‘grade’, ‘transparency of examination requirements’ and ‘overcommitment’ were significantly and positively associated with suicidal ideation; ‘reward’ was significantly and negatively associated with suicidal ideation. The aspects ‘learning group’, ‘performance expectation from lecturers’ and ‘support in exam preparations’ were no longer associated with suicidal ideation.

By including the scales of the Achievement Goal Inventory, the significant association of model 5 remained almost unchanged. Thus, no significant associations with suicidal ideation were found for the Achievement Goal Inventory scales.

The highest proportion of variance was explained by model 3, followed by model 5, model 1 and model 6, while model 2 and model 4 contributed only very slightly to variance explanation (Table 4).

### 3.5. Potential Risk Factors Associated with Suicide Risk

The demographic variables in model 1 did not reveal any significant associations with suicide risk.

In model 2, less ‘transparency of study requirements’ showed a significantly positive association with suicide risk. The demographic variables remained without significant associations.

After adding social climate variables (model 3), ‘transparency of study requirements’ remained significant. In addition, there were significant negative associations with suicide risk in ‘time of study choice’ and ‘learning group’, as well as significant positive associations with suicide risk in ‘transparency of examination requirements’ and ‘performance expectation from the lecturers’.

None of the additional variables included in model 4 were significantly associated with increased suicide risk. Moreover, ‘learning group’ was no longer a significant predictor for suicide risk. The associations of ‘time of study choice’, ‘transparency of study requirements’ and ‘examination requirements’ and ‘performance expectation from the lecturers’ with suicide risk remained significant.

After including the ERI scales in model 5, of the previous variables, only ‘transparency of study requirements and examination requirements’ remained significantly associated with suicide risk. Additionally, the ERI variables ‘reward’ and ‘overcommitment’ were significantly associated with suicide risk, with higher reward being associated with a lower probability of suicide risk, while higher ‘overcommitment’ increased this likelihood.

Adding the Achievement Goal Inventory in model 6, the associations of ‘transparency of study and examination requirements’, as well as the ERI scales ‘reward’ and ‘overcommitment’, with suicide risk showed no change. The newly added Achievement Goal Inventory items revealed no significant associations with suicide risk.

The highest proportion of variance was explained by model 5, followed by model 3, model 2 and model 1, while model 4 and model 6 were almost negligible in terms of variance explanation (Table 5).

## 4. Discussion

In the past years, several studies from different countries provide evidence that veterinary medical students show a significantly increased risk of depression, suicidal ideation and death by suicide [7,8,9,10,11,12,13,14,16,19,25,27,65,66]. A study on depression, suicidal ideation and suicide risk among German veterinary students identified depressive symptoms in 49.9% of the students, suicidal ideation in 19.9% and an increased suicide risk in 24.0%. Thus, veterinary students showed a twenty-twofold-higher risk of depression, a fourfold-higher risk of suicidal ideation and a fourfold-higher suicide risk compared to the German general population of the same age range [17]. The study presented is the first study on demographic and study-related risk factors for depression, suicidal ideation and suicide risk among German veterinary students and revealed low ‘reward’ and high ‘overcommitment’ as the most important predictors for depression, suicidal ideation and suicide risk. Moreover, ‘time of study choice’, ‘grade’, ‘learning group’, ‘peer competition’ and ‘performance expectation from lecturers’ as well as ‘transparency of study requirements’ and ‘transparency of examination requirements’ were also observed to be significantly associated with depression, suicidal ideation and suicide risk.

Numerous studies in various geographical regions have mentioned different specific risk factors in veterinary students, such as the time-consuming workload and continuous examination load within the undergraduate veterinary program, chronic sleep deprivation, problems with family or personal relationships, perception of difficulty fitting in with other students, competition between peers, unclear instructor expectations, feeling being behind in studies, concerns about falling short the academic outcomes, a hidden fear of failing, not having enough time for friends and leisure activities and a shortcoming of private life in order to have more time to study [7,8,11,12,13,14,16,25,26,27,28,30,31]. For German veterinary students, such data have been lacking so far.

Therefore, the present study aimed to explore potential demographic and study-related risk factors as potential predictors of depression, suicidal ideation and suicide risk in a representative sample of German veterinary students.

With the factors used, the highest variance explanation was found for depression. Overall, demographic factors contributed only very little to variance explanation. However, most of the models of the regression analysis showed a negative association of the ‘time of study choice’ with depression as well as a positive association of ‘grade’ with depression. Female students in particular, who are overrepresented in veterinary medicine studies, usually make the decision to study veterinary medicine very early in life and cite a love of animals as an important factor in their decision [28,67]. However, the reality experienced during their studies could become a burden for these students. Additionally, students who were already high-achievers in school and continue their pursuit of excellence during their course of study might find it very stressful when they perform poorly for the first time at university [28,29,30]. However, such a perceived loss of control regarding academic performance expectations poses a higher risk of depression, especially for high-performing students [68]. Among the factors listed under social climate that contributed to a higher extent to variance explanation, ‘learning group’, ‘peer competition’ and ‘performance expectation from lecturers’ were significantly associated with depression. The factor ‘learning group’ was negatively related to the probability of depression. Several studies mentioned that professional undergraduate human medical programs as well as veterinary programs are often intensive and demanding, with a time-consuming workload and a continuous examination load [7,8,11,12,13,14,16,25,26,27,28,29,30,31]. Therefore, student–student relationships might have a positive impact on students’ coping with work, as collaboration seems to provide the opportunity to help each other understand difficult concepts [69]. The feeling of a common understanding of the situation and being “in the same boat” serves as an informal peer support mechanism [11]. Moreover, the development of close, collaborative and supportive relationships with fellow students can serve as a protective factor in terms of stress [65,70]. That ‘peer competition’ could be a potential predictor of depression is supported by a study on veterinary students, in which a high correlation was found between stress and depression, with high levels of competition between students being identified as a stress-inducing factor [11]. Furthermore, our finding that ‘performance expectation from lecturers’ could be a relevant predictor of depression is confirmed by the results of other studies [7,27]. The three ERI subscales, ‘effort’, ‘reward’ and ‘overcommitment’, contributed to the highest extent of the explained variance and are therefore the potentially most important predictors investigated of depression. Thereby, ‘reward’ was strongly negatively related, while ‘effort’ as well as ‘overcommitment’ were strongly positively related to the probability of depression. These associations were also confirmed in other studies among workers [71,72], physicians [73], veterinarians [74,75] and students [54,57]. Although these relationships were found for both non-medical and medical students, the associations between the ERI components and depression were more pronounced in medical students [54]. Medical students reported that the medical school curriculum and environment are principal sources of distress [13,31]. Veterinary students stated that, among other things, time spent studying and a heavy workload were perceived as the most distressing [7,8]. Additionally, the lack of time can lead to distress when students are trying to balance all aspects of their lives [70]. A chronic imbalance between job demands and the resources one has to complete the job can lead to unresolvable long-term work-related stress [17,76]. As a result, this can lead to exhaustion and burnout [17,70,76,77,78]. Other studies found that veterinary students who feel behind in studies worry about not being as smart as others, and those perceived not to fit in with colleagues were statistically more likely to experience anxiety and depression symptoms [66,70]. Furthermore, if these feelings and behaviors persist during studies, they can result in a poorer work–life balance, stress, anxiety, social isolation and depression [13,26,27]. Although medical students show strong effort in coping with a high study load and invest time and energy into learning, they may perceive fewer learning outcomes than achievements and rewards, less self-esteem and feel less respected in the academic environment [52,53,57,79]. It can be assumed that the same applies to veterinary students. Since the results for veterinary students in Germany show that the imbalance between ‘effort’ and ‘reward’ primarily affects female students, it can be assumed that the high association between ‘effort’ and ‘reward’ with depression is of higher relevance for female students. Overcommitment is defined as the tendency of individuals to show excessive work-related engagement [55,56,80]. Overcommitment is a specific personal coping strategy that predisposes individuals to excessive work-related effort [52,75,80] and can thus be separately considered as an intrinsic component in the ERI model [75]. The high positive association of ‘overcommitment’ with depression found is also confirmed by several studies that revealed that among university students, increased overcommitment is related to an increased vulnerability to illness, chronic stress, burnout, anxiety and depressive symptoms [54,55,56,57]. In employees, overcommitment can occur when, among other things, the demands of the job exceed the employee’s competencies, there is a strong need for recognition and a fear of failure at work, an increased competitive urge, an increased perception of time pressure or an inability to distance oneself from work demands [75]. This also results in an increased risk of depression, emotional exhaustion, burnout and anxiety for employees [75]. Students of veterinary medicine also showed a high willingness to perform and reported competition among peers, concerns about academic performance, a feeling of being behind in studies, concerns about failing to meet academic expectations, a hidden fear of failing and associated social isolation and stress [7,8,17,18,20,21,23,29].

A lower amount of variance explanation by the factors included was found for suicidal ideation. The variables ‘transparency of study and examination requirements’ used in this study contributed only very little to the variance explained in suicidal ideation. Nevertheless, ‘transparency of examination requirements’ was significantly positively associated with suicidal ideation in most models of the regression analysis and could be a potentially relevant predictor for suicidal ideation. Actually, it could be assumed that ‘transparency of examination requirements’ is associated with a reduction in suicidal ideation. When situations are no longer clearly transparent, a tendency to develop a negative attitude towards one’s own performance can occur [81]. The requirements of an examination should therefore be transparent [81]. This is in line with recommendations for improving mental health in veterinary students, which suggest clarifying specific learning objectives [30]. However, transparency must be kept very specific [81]. While transparency regarding requirements can contribute to provide clarity and reduce anxiety, it does not increase competency in the examination [81]. The results of a study among veterinary students revealed that two-thirds felt overwhelmed by the heavy workload and more than 70% reported being worried about failing examinations and not being able to graduate [25]. Finally, it can be assumed that the positive association between ‘transparency of examination requirements’ and suicidal ideation results from the transparency of the large number of examination objectives. Most demographic variables also accounted for only a small amount of the variance explained. However, the factor ‘grade’ showed a significant positive association with the probability of suicidal ideation in all models of the regression analysis and was indicated as a potentially relevant predictor of suicidal ideation. This study indicated that the better the ‘grade’, the higher the association with an increased likelihood of suicidal ideation. This stands in contrast to the results of other studies that demonstrate a strong association between poor grades of school and college students with suicidal ideation or suicide risk among adolescents and in young adults [82,83,84,85,86]. In the case of veterinary students, the relationship between an excellent average grade in the university admission qualification and suicidal ideation might be explained by the fact that veterinary students belong to the so-called high-achieving elite performers [29]. As in other countries, the admission requirements for veterinary medicine studies are very high in Germany. One of the main criteria for admission is an extremely good average grade in the university admission qualification. Consequently, a large proportion of places are awarded to students with the best grades. This leads to homogeneous student cohorts that are usually highly intelligent and gifted [87]. In order to still be better than their peers and to avoid negative social comparison, some students employ a highly competitive behavior that decreases cooperation between students [29]. Struggling students perceive difficulty fitting in with other students [8]. Thereby, the sense of belongingness is central for veterinary students [28]. Joiner’s interpersonal psychological theory of suicide (IPTS) postulates that the feeling of not belonging to an appreciative group (thwarted belongingness) is a core component in the development of suicidal ideation [28,88,89]. The significant association between ‘grade’ and suicidal ideation identified in our study may therefore be related to the personality traits of the students as high-achievers [28,29,30]. Even though most of the variables of “social climate” were not significant at all or not consistently, they contributed the highest amount to the explained variance. Hereby, the factor ‘learning group’ was negatively and the factor ‘performance expectation from lecturers’ positively associated with the probability of suicidal ideation. As already mentioned, a sense of belonging may serve as a protective factor against suicidal ideation in veterinary students [28,88,89]. That ‘learning group’ could be a relevant predictor of suicidal ideation is supported by a study that reported the strong relation between getting to know other students and finding learning groups with students’ integration into the university [90]. A study among first-year university students confirms the finding that ‘performance expectation from lecturers’ could serve as a relevant predicator for suicidal ideation [69]. This study indicated a positive association between a good student–lecturer relationship, including the possibility to discuss problems and knowledge of lecturers’ expectations, with psychological well-being and life satisfaction [69]. However, both factors were no longer significant when the ERI variables were added. Although the ERI subscale ‘effort’ was not significant, all ERI subscales contributed to a high extent to the explained variance. The ‘reward’ subscale was significantly negatively related to the likelihood of suicidal ideation, while the ‘overcommitment’ subscale was significantly positively associated with suicidal ideation. Other studies investigating the associations between ‘reward’ and ‘overcommitment’ with adverse health effects, including poor self-rated health [52,54], musculoskeletal disorders and cardiovascular as well as metabolic diseases [52], psychological distress [57,75], burnout [55], anxiety and depressive symptoms [54,72] and suicidal ideation [91,92] support our findings that ‘reward’ and ‘overcommitment’ were identified as the most relevant predictors of suicidal ideation.

The lowest variance explanation with the factors used was found for suicide risk. Although the aspects assessed with the variables ‘transparency of study and examination requirements’ made little contribution to the explained variance of suicide risk, the aspect ‘transparency of study requirements’ was consistently and significantly negatively associated with suicide risk. In contrast, the factor ‘transparency of examination requirements’ was significantly positively related to suicide risk in most models of the regression analysis. Several studies among medical students and veterinary students indicated the high work- and examination load as potential risk factors for poor mental health [7,8,12,13,27,29,30,35]. Concerns about workload were exacerbated when students were not sure which material was “core knowledge” and which was offered only for additional interest [30]. A study among veterinary students showed a significant positive association between unclear expectations and anxiety [7]. Anxiety can become generalized [81]. If students receive no support, their anxiety and fear of failing can turn into helplessness and even lead to suicide [81]. Since clear expectations may reduce anxiety levels for students [7], it can be assumed that ‘transparency of study requirements’ could be a potentially important predictor of suicide risk. As with suicidal ideation, it can be presumed that the positive association between ‘transparency of examination requirements’ and suicide risk may reflect concerns about the large number of examination objectives. Consequently, ‘transparency of examination requirements’ could serve as a relevant predictor of suicidal ideation. The ERI scales showed the highest contribution to the explained variance. Since the ’effort’ scale was not significant, the ‘reward’ and ‘overcommitment’ scales were the main contributors, with ‘reward’ being significantly negatively and ‘overcommitment’ being significantly positively associated with suicide risk. Thus, ‘reward’ and ‘overcommitment’ can be considered as potentially the most relevant predictors of suicidal risk. A recent study among German veterinarians that found significant relationships between low reward and high overcommitment and suicide risk confirms our findings [74].

Based on these findings, it is recommended to develop suitable prevention and intervention measures at veterinary medical schools. A variety of recommendations are given in other studies, such as training on coping strategies, resilience and study-related stress management, promoting good relationships with instructors and peers, implementation of mentoring programs and psychosocial support services [57] as well as the development and provision of educational resources in advance of classes and development of defined learning objectives that transparently show whether the learning objectives are mandatory or supplementary [30]. Considering the specific personality traits of veterinary students as high-achievers [28,29,30], it seems especially important to develop customized support services. Programs with a positive impact on veterinary students’ resilience should be offered from the beginning of the program and cover various resilience-building aspects, including personal mental health and well-being, coping strategies, learning strategies and communication and business administration [9,93,94]. Further intervention programs strengthening resilience could be established at veterinary schools. Then, longitudinal studies would be required to assess their effectiveness.

### Limitations

Several limitations of this study must be pointed out. First, since the response rate is low, the results may not be generalized to the entire population of veterinary students in Germany. Second, no a priori power analysis was conducted. However, the results of a post hoc analysis indicated that the sample size of the study was sufficient to detect significant effects. Third, due to the cross-sectional nature of this study, the results may not be interpreted as trajectories of mental health problems across the study program but represent findings from different cohorts of students. Fourth, as among veterinary students [95], women are overrepresented in our study. The higher proportion of female and lower proportion of male veterinary students in our study compared to the entire population of German veterinary students limit the generalizability of the results for male German veterinary students. Fifth, compared to men, women tend to report mental distress more often [96,97,98]. The voluntary nature of participation therefore carries the risk of a bias due to an increased proportion of participants suffering from mental health issues and possibly having personal experiences related to the topic of this study. Sixth, item 9 of the PHQ-9 was used to assess suicidal ideation. Compared to clinical interviews, the use of a single-item assessment might not reflect different aspects of suicidal ideation. Since the method used is internationally established for screening in research studies [99,100] and comparative data were collected in a similar way, we decided in favor of it. Seventh, the perceived workload assessed using the effort–reward imbalance questionnaire represents self-reported subjective perceptions. Since this is not a direct comparison of the actual workload, the nature of individual perception limits the comparability of the results. Eighth, the survey was conducted in an online format without an individual access code. Therefore, it is possible that participants completed the survey more than once. However, due to the length and duration of the survey, this is considered very unlikely. Ninth, several studies have shown that students’ mental health has worsened due to the restrictions caused by the COVID-19 pandemic [101,102,103]. Since the data were collected prior to the pandemic, the current mental health of veterinary students might be even worse than the study results suggest. Tenth, comparisons of our results with the results of other studies cited should be interpreted with caution due to different methods and instruments used.

## 5. Conclusions

In conclusion, it can be stated that the variance explanation was strongest overall for depression, less strong for suicidal ideation and least strong for suicide risk. The ERI variables ‘effort’, ‘reward’ and ‘overcommitment’ proved to be the most important predictors for depression. For suicidal ideation and suicide risk, the strongest predictors were ‘reward’ and ‘overcommitment’. Additionally, ‘time of study choice’, ‘grade’, ‘learning group’, ‘peer competition’, and ‘performance expectation from lecturers’ as well as ‘transparency of study requirements’ and ‘transparency of examination requirements’ were found to be further potentially relevant predictors, although they made a smaller contribution to the explained variance. Since this is a cross-sectional study, assumptions about potential shifts in depression, suicidal ideation and suicide risk among students over the entire study program cannot be made. Conducting a longitudinal study to investigate an entire cohort of German veterinary students is therefore recommended.

Overall, the development of customized prevention and intervention measures that cover several strategies for strengthening resilience, positive relationships with peers and instructors and for coping with learning and examination requirements is recommended, starting from the beginning of the program.

## Figures and Tables

**Table 1:** Demographic characteristics of the sample.

	Female		Male		Total	
	n	%	n	%	N	%
**Time of study choice**						
Before primary school	109	13.2	4	4.8	113	12.4
During primary school	140	16.9	14	16.7	154	16.9
During lower secondary level	103	12.4	15	17.9	118	12.9
During upper secondary level	127	15.3	13	15.5	140	15.4
At the time of upper secondary school leaving examination	77	9.3	10	11.9	87	9.5
During an orientation period after leaving secondary school	126	15.2	12	14.3	138	15.1
During vocational training/during another study program	125	15.1	13	15.5	138	15.1
**Semester**						
1st	107	12.9	8	9.5	115	12.6
3rd	198	23.9	17	20.2	215	23.6
5th	202	24.4	22	26.2	224	24.6
7th	146	17.6	11	13.1	157	17.2
9th	69	8.3	13	15.5	82	9.0
11th	106	12.8	13	15.5	119	13.0
**Vocational training**	186	22.5	15	17.9	201	22.1
**Employment income**	67	8.1	14	17.1	81	8.9

**Table 2:** Study-related factors.

	Female			Male				Total	
	n	m	sd	n	m	sd	N	M	SD
**Requirements**									
Transparency of study requirements	781	3.91	1.22	79	3.84	1.20	860	3.91	1.21
Transparency of examination requirements	799	3.59	1.25	80	3.70	1.43	879	3.60	1.27
**Social climate**									
Peer competition	824	2.96	1.47	84	2.87	1.40	908	2.96	1.47
Students’ working atmosphere	825	4.22	1.26	83	4.31	1.24	908	4.23	1.26
Learning group	824	3.24	1.65	84	2.96	1.63	908	3.21	1.65
Performance expectation from lecturers	821	4.75	1.18	84	4.44	1.32	905	4.72	1.19
Working atmosphere with lecturers	813	3.89	1.10	83	4.05	1.02	896	3.90	1.09
**Satisfaction with support and supervision provided by the lecturers**									
Teaching learning strategies	710	2.87	1.41	70	3.10	1.21	780	2.89	1.39
Support with learning difficulties	618	2.55	1.24	64	2.80	1.12	682	2.58	1.23
Support in practical skills	749	3.47	1.36	78	3.45	1.39	827	3.47	1.37
Support in exam preparations	717	2.76	1.23	74	2.97	1.16	791	2.78	1.23
Receiving feedback	657	2.69	1.31	70	2.63	1.32	727	2.69	1.31
Lecturers’ contactibility	683	3.76	1.46	71	4.07	1.25	754	3.79	1.44
**Effort–Reward Imbalance Questionnaire**									
Effort	751	9.32	1.54	76	8.70	2.07	872	9.26	1.60
Reward	677	15.44	3.07	79	16.37	3.36	756	15.54	3.11
Overcommitment	820	14.42	3.63	81	12.48	3.66	901	14.25	3.67
Effort–Reward Ratio	641	1.21		73	1.06		714	1.19	
**Achievement Goal Inventory**									
Outcome Goals	820	4.66	1.51	84	4.70	1.54	904	4.66	1.51
Ability Goals	826	3.94	1.48	84	4.02	1.58	910	3.95	1.49
Learning/Challenge Goals	825	4.79	1.04	80	4.98	1.07	905	4.81	1.04
Normative Goals	825	2.30	1.33	84	2.59	1.44	909	2.32	1.34

**Table 3:** Hierarchical logistic regression analyses of factors associated with depression.

Characteristics	Model 1		Model 2		Model 3		Model 4		Model 5		Model 6	
	OR (95% CI)	*p*	OR (95% CI)	*p*	OR (95% CI)	*p*	OR (95% CI)	*p*	OR (95% CI)	*p*	OR (95% CI)	*p*
Gender	1.90 (0.92–3.93)	0.083	1.93 (0.91–4.07)	0.334	1.65 (0.75–3.64)	0.214	1.50 (0.67–3.38)	0.326	0.91 (0.34–2.40)	0.919	0.99 (0.36–2.71)	0.990
Age	1.10 (1.01–1.19)	0.028	1.08 (0.99–1.18)	0.077	1.08 (0.98–1.18)	0.127	1.06 (0.96–1.16)	0.246	1.06 (0.95–1.19)	0.318	1.05 (0.94–1.18)	0.400
Time of study choice	0.89 (0.80–0.98)	0.025	0.89 (0.80–0.98)	0.025	0.86 (0.77–0.96)	0.008	0.86 (0.77–0.96)	0.007	0.88 (0.77–1.01)	0.056	0.86 (0.75–0.98)	0.030
Semester	0.96 (0.82–1.12)	0.610	0.89 (0.76–1.06)	0.198	0.92 (0.77–1.09)	0.337	0.89 (0.74–1.07)	0.208	0.93 (0.74–2.16)	0.497	0.91 (0.71–1.14)	0.402
Grade	0.53 (0.33–0.85)	0.009	0.52 (0.32–0.85)	0.009	0.51 (0.30–0.86)	0.014	0.54 (0.31–0.93)	0.025	0.53 (0.27–1.04)	0.066	0.57 (0.28–1.16)	0.124
Vocational training	0.69 (0.37–1.28)	0.238	0.73 (0.38–1.38)	0.334	0.73 (0.37–1.47)	0.382	0.78 (0.38–1.60)	0.503	0.96 (0.41–2.24)	0.919	1.11 (0.46–2.67)	0.814
Employment income	1.025 (0.84–1.26)	0.808	0.99 (0.812–1.23)	0.993	0.95 (0.76–1.19)	0.676	0.96 (0.76–1.20)	0.698	0.94 (0.72–1.24)	0.663	0.92 (0.69–1.21)	0.552
**Requirements**												
Transparency of study requirements			0.89 (0.71–1.11)	0.314	0.98 (0.76–1.25)	0.848	0.97 (0.75–1.25)	0.809	1.14 (0.83–1.56)	0.435	1.06 (0.76–1.47)	0.741
Transparency of examination requirements			0.79 (0.63–0.98)	0.031	0.85 (0.66–1.09)	0.214	0.91 (0.70–1.81)	0.476	1.05 (0.76–1.46)	0.758	1.03 (0.74–2.44)	0.876
**Social climate**												
Peer competition					1.34 (1.15–1.58)	<0.001	1.33 (1.13–1.57)	<0.001	1.14 (0.93–1.39)	0.197	1.08 (0.88–1.33)	0.481
Students’ working atmosphere					0.84 (0.69–1.02)	0.080	0.86 (0.70–1.05)	0.126	0.84 (0.66–1.07)	0.164	0.81 (0.64–1.04)	0.104
Learning group					0.85 (0.74–0.97)	0.016	0.84 (0.73–0.96)	0.013	0.85 (0.72–0.99)	0.049	0.86 (0.73–1.03)	0.097
Performance expectation from lecturers					1.53 (1.24–1.88)	<0.001	1.47 (1.19–1.82)	<0.001	1.16 (0.89–1.50)	0.277	1.13 (0.86–1.48)	0.371
Working atmosphere with lecturers					0.94 (0.75–1.19)	0.610	0.99 (0.78–1.29)	0.991	1.11 (0.82–1.50)	0.501	1.08 (0.79–1.49)	0.612
**Satisfaction with support and supervision provided by the lecturers**												
Teaching learning strategies							0.92 (0.73–1.15)	0.463	0.86 (0.65–1.12)	0.261	0.84 (0.64–1.10)	0.201
Support with learning difficulties							1.06 (0.79–1.42)	0.687	1.24 (0.88–1.75)	0.211	1.20 (0.85–1.68)	0.295
Support in practical skills							1.07 (0.87–1.32)	0.541	1.01 (0.78–1.29)	0.977	1.03 (0.79–1.34)	0.840
Support in exam preparations							0.73 (0.56–0.95)	0.021	0.75 (0.54–1.04)	0.085	0.79 (0.57–1.09)	0.158
Receiving feedback							0.98 (0.80–1.21)	0.880	1.03 (0.80–1.31)	0.835	1.08 (0.84–1.40)	0.555
Lecturers’ contactibility							0.99 (0.82–1.19)	0.902	1.09 (0.87–1.36)	0.467	1.06 (0.84–1.34)	0.621
**Effort–Reward Imbalance Questionnaire**												
Effort									1.35 (1.09–1.66)	0.005	1.37 (2.11–1.70)	0.004
Reward									0.80 (0.71–0.89)	<0.001	0.79 (0.70–0.899)	<0.001
Overcommitment									1.40 (1.27–1.54)	<0.001	1.39 (1.26–1.54)	<0.001
**Achievement Goal Inventory**												
Outcome Goals											0.98 (0.77–1.25)	0.869
Ability Goals											1.57 (1.20–2.06)	<0.001
Learning/Challenge Goals											0.85 (0.60–1.18)	0.329
Normative Goals											0.92 (0.72–1.18)	0.519
**Model**	Adjusted R^2^ = 0.054		Adjusted R^2^ = 0.099		Adjusted R^2^ = 0.26		Adjusted R^2^ = 0.28		Adjusted R^2^ = 0.55		Adjusted R^2^ = 0.57	
	*p* < 0.016		*p* < 0.001		*p* < 0.001		*p* < 0.001		*p* < 0.001		*p* < 0.001	

Notes. Gender: 1 = female, 2 = male; vocational training: 0 = no, 1 = yes; employment income 0 = no, 1 = yes; grade: inverse association, as in the German grading system a lower number represents a better grade.

**Table 4:** Hierarchical logistic regression analyses of factors associated with suicidal ideation.

Characteristics	Model 1		Model 2		Model 3		Model 4		Model 5		Model 6	
	OR (95% CI)	*p*	OR (95% CI)	*p*	OR (95% CI)	*p*	OR (95% CI)	*p*	OR (95% CI)	*p*	OR (95% CI)	*p*
Gender	1.48 (0.55–4.02)	0.438	1.55 (0.57–4.22)	0.389	1.22 (0.43–3.48)	0.705	1.15 (0.39–3.36)	0.797	0.71 (0.22–2.22)	0.554	0.79 (0.24–2.56)	0.692
Age	1.06 (0.96–1.17)	0.251	2.05 (0.95–1.17)	0.306	1.04 (0.93–1.15)	0.535	1.01 (0.90–1.13)	0.845	0.98 (0.87–1.10)	0.736	0.99 (0.88–1.12)	0.879
Time of study choice	0.94 (0.83–1.07)	0.339	0.94 (0.83–1.06)	0.302	0.91 (0.80–1.04)	0.176	0.92 (0.80–1.05)	0.221	0.96 (0.83–1.11)	0.569	0.96 (0.83–1.12)	0.639
Semester	1.04 (0.86–1.25)	0.713	1.02 (0.84–1.23)	0.870	1.06 (0.86–1.30)	0.597	1.01 (0.81–1.26)	0.908	1.09 (0.86–1.389)	0.488	1.07 (0.84–1.37)	0.586
Grade	0.42 (0.23–0.79)	0.007	0.42 (0.23–0.79)	0.007	0.41 (0.21–0.77)	0.006	0.43 (0.22–0.84)	0.014	0.44 (0.22–0.89)	0.022	0.48 (0.24–0.98)	0.044
Vocational training	1.05 (0.47–2.33)	0.914	1.02 (0.46–2.28)	0.956	1.11 (0.47–2.63)	0.811	1.24 (0.51–3.03)	0.636	1.60 (0.62–4.11)	0.329	1.57 (0.60–4.08)	0.357
Employment income	1.15 (0.90–1.47)	0.257	1.14 (0.89–1.46)	0.292	1.13 (0.86–1.47)	0.383	1.12 (0.85–1.48)	0.410	1.14 (0.85–1.52)	0.381	1.14 (0.85–1.53)	0.382
**Requirements**												
Transparency of study requirements			0.74 (0.56–0.98)	0.034	0.80 (0.60–1.09)	0.153	0.82 (0.60–1.12)	0.217	0.89 (0.65–1.24)	0.512	0.88 (0.63–1.22)	0.437
Transparency of examination requirements			1.16 (0.88–1.53)	0.297	1.46 (1.08–1.99)	0.016	1.51 (1.10–2.07)	0.011	1.72 (1.22–2.43)	0.002	1.76 (1.24–2.51)	0.002
**Social climate**												
Peer competition					1.18 (0.98–1.43)	0.084	1.19 (0.97–1.45)	0.087	1.03 (0.83–1.27)	0.784	0.97 (0.77–1.22)	0.809
Students’ working atmosphere					0.86 (0.68–1.08)	0.193	0.89 (0.70–1.13)	0.341	0.94 (0.72–1.21)	0.627	0.91 (0.70–1.18)	0.483
Learning group					0.85 (0.72–1.00)	0.050	0.82 (0.69–0.97)	0.022	0.83 (0.69–1.01)	0.052	0.86 (0.71–1.05)	0.137
Performance expectation from lecturers					1.45 (1.09–1.92)	0.010	1.14 (1.06–1.88)	0.017	1.19 (0.87–1.64)	0.274	1.16 (0.84–1.60)	0.364
Working atmosphere with lecturers					0.71 (0.53–0.95)	0.019	0.78 (0.58–1.07)	0.119	0.89 (0.59–1.16)	0.276	0.79 (0.56–1.11)	0.167
**Satisfaction with support and supervision provided by the lecturers**												
Teaching learning strategies							1.04 (0.77–1.39)	0.823	0.98 (0.72–1.35)	0.909	0.99 (0.72–1.36)	0.943
Support with learning difficulties							1.19 (0.81–1.75)	0.362	1.31 (0.88–1.95)	0.191	1.22 (0.81–1.84)	0.338
Support in practical skills							0.83 (0.64–1.09)	0.174	0.84 (0.63–1.12)	0.232	0.89 (0.67–1.19)	0.444
Support in exam preparations							0.69 (0.49–0.96)	0.030	0.74 (0.52–1.04)	0.085	0.76 (0.54–1.09)	0.133
Receiving feedback							1.06 (0.81–1.37)	0.683	1.12 (0.85–1.47)	0.436	1.14 (0.86–1.51)	0.350
Lecturers’ contactibility							0.91 (0.72–1.16)	0.456	0.97 (0.76–1.25)	0.827	0.97 (0.75–1.26)	0.836
**Effort–Reward Imbalance Questionnaire**												
Effort									1.03 (0.81–1.31)	0.820	1.05 (0.82–1.34)	0.721
Reward									0.83 (0.73–0.95)	0.005	0.80 (0.70–0.92)	0.002
Overcommitment									1.27 (1.28–1.42)	<0.001	1.24 (1.11–1.39)	<0.001
**Achievement Goal Inventory**												
Outcome Goals											1.18 (0.90–1.53)	0.223
Ability Goals											1.21 (0.91–1.60)	0.188
Learning/Challenge Goals											0.93 (0.66–1.31)	0.687
Normative Goals											0.96 (0.72–1.26)	0.687
**Model**	Adjusted R^2^ = 0.046		Adjusted R^2^ = 0.064		Adjusted R^2^ = 0.185		Adjusted R^2^ = 0.215		Adjusted R^2^ = 0.330		Adjusted R^2^ = 0.364	
	*p* = 0.09		*p* = 0.05		*p* < 0.001		*p* < 0.001		*p* < 0.001		*p* < 0.001	

Notes. Gender: 1 = female, 2 = male; vocational training: 0 = no, 1 = yes; employment income 0 = no, 1 = yes; grade: inverse association, as in the German grading system a lower number represents a better grade.

**Table 5:** Hierarchical logistic regression analysis of factors associated with suicide risk.

Characteristics	Model 1		Model 2		Model 3		Model 4		Model 5		Model 6	
	OR (95% CI)	*p*	OR (95% CI)	*p*	OR (95% CI)	*p*	OR (95% CI)	*p*	OR (95% CI)	*p*	OR (95% CI)	*p*
Gender	1.37 (0.57–3.30)	0.479	1.49 (0.61–3.63)	0.378	1.29 (0.51–3.24)	0.586	1.15 (0.45–2.93)	0.769	0.75 (0.27–2.07)	0.576	0.77 (0.27–2.20)	0.624
Age	1.05 (0.96–1.15)	0.250	1.04 (0.95–1.15)	0.390	1.04 (0.95–1.15)	0.401	1.03 (0.93–1.13)	0.593	1.02 (0.92–1.13)	0.682	1.02 (0.92–1.13)	0.741
Time of study choice	0.91 (0.81–1.02)	0.087	0.89 (0.80–1.1)	0.062	0.88 (0.78–0.99)	0.040	0.88 (0.78–0.99)	0.038	0.90 (0.79–1.03)	0.113	0.90 (0.79–1.03)	0.129
Semester	0.98 (0.83–1.17)	0.858	0.96 (0.80–1.15)	0.689	1.01 (0.83–1.21)	0.984	0.98 (0.81–1.19)	0.856	1.01 (0.82–1.25)	0.909	1.03 (0.83–1.28)	0.782
Grade	0.59 (0.34–1.02)	0.059	0.60 (0.34–1.05)	0.073	0.59 (0.33–1.06)	0.079	0.62 (0.34–1.12)	0.111	0.59 (0.32–2.12)	0.106	0.64 (0.34–1.21)	0.173
Vocational training	0.86 (0.42–1.76)	0.858	0.83 (0.40–1.73)	0.625	0.82 (0.39–1.74)	0.607	0.86 (0.40–1.86)	0.705	1.03 (0.45–2.32)	0.951	1.05 (0.46–2.39)	0.908
Employment income	1.16 (0.92–1.45)	0.210	1.14 (0.91–1.44)	0.256	1.13 (0.89–1.44)	0.309	1.13 (0.88–1.44)	0.339	1.14 (0.88–1.48)	0.317	1.13 (0.86–1.47)	0.378
**Requirements**												
Transparency of study requirements			0.62 (0.48–0.81)	<0.001	0.63 (0.48–0.83)	0.001	0.64 (0.48–0.85)	0.002	0.66 (0.49–0.87)	0.006	0.65 (0.48–0.88)	0.005
Transparency of examination requirements			1.28 (0.99–1.65)	0.059	1.53 (1.15–2.03)	0.003	1.54 (1.15–2.06)	0.003	1.87 (1.35–2.58)	<0.001	1.89 (1.37–2.63)	<0.001
**Social climate**												
Peer competition					1.10 (0.93–1.31)	0.277	1.09 (0.92–1.30)	0.304	0.92 (0.76–1.12)	0.422	0.90 (0.74–1.11)	0.340
Students’ working atmosphere					1.02 (0.82–1.27)	0.851	1.03 (0.83–1.29)	0.759	1.14 (0.89–1.45)	0.293	1.12 (0.88–1.42)	0.363
Learning group					0.86 (0.74–0.99)	0.043	0.86 (0.74–1.01)	0.055	0.88 (0.74–1.03)	0.111	0.87 (0.73–1.03)	0.110
Performance expectation from lecturers					1.39 (1.09–1.78)	0.008	1.36 (1.06–1.75)	0.014	1.14 (0.87–1.50)	0.356	1.12 (0.85–1.48)	0.428
Working atmosphere with lecturers					0.79 (0.61–1.12)	0.067	0.82 (0.62–1.08)	0.151	0.88 (0.65–1.81)	0.386	0.87 (0.64–1.17)	0.348
**Satisfaction with support and supervision provided by the lecturers**												
Teaching learning strategies							0.97 (0.75–1.33)	0.793	0.91 (0.69–1.19)	0.493	0.91 (0.67–1.20)	0.496
Support with learning difficulties							0.97 (0.70–1.35)	0.870	1.05 (0.75–1.47)	0.797	1.05 (0.74–1.48)	0.784
Support in practical skills							1.00 (0.79–1.26)	0.998	0.99 (0.78–1.28)	0.981	1.01 (0.78–1.30)	0.948
Support in exam preparations							0.88 (0.66–1.18)	0.392	0.97 (0.72–1.31)	0.847	0.96 (0.71–1.31)	0.793
Receiving feedback							1.10 (0.88–1.38)	0.396	1.19 (0.94–1.52)	0.150	1.21 (0.95–1.54)	0.132
Lecturers’ contactibility							0.93 (0.75–1.15)	0.508	0.97 (0.77–1.21)	0.781	0.96 (0.77–1.21)	0.744
**Effort–Reward Imbalance Questionnaire**												
Effort									1.14 (0.92–1.41)	0.223	1.17 (0.94–1.45)	0.165
Reward									0.79 (0.71–0.89)	<0.001	0.77 (0.68–0.87)	<0.001
Overcommitment									1.19 (1.08–1.30)	<0.001	1.18 (1.07–1.29)	<0.001
**Achievement Goal Inventory**												
Outcome Goals											1.04 (0.82–1.31)	0.755
Ability Goals											1.10 (0.86–1.41)	0.443
Learning/Challenge Goals											1.21 (0.88–1.65)	0.244
Normative Goals											0.92 (0.72.1.18)	0.520
**Model**	Adjusted R^2^ = 0.034		Adjusted R^2^ = 0.081		Adjusted R^2^ = 0.152		Adjusted R^2^ = 0.159		Adjusted R^2^ = 0.300		Adjusted R^2^ = 0.311	
	*p* = 0.23		*p* = 0.007		*p* < 0.001		*p* < 0.001		*p* < 0.001		*p* < 0.001	

Notes. Gender: 1 = female, 2 = male; vocational training: 0 = no, 1 = yes; employment income 0 = no, 1 = yes; grade: inverse association, as in the German grading system a lower number represents a better grade.

## Data Availability

The data presented in this study are available on reasonable request from the corresponding author.

## Abbreviations

The following abbreviations are used in this manuscript:

BAföG	Federal Training Assistance Act
TAppV	German Veterinary Medical Licensure Law
PHQ-9	Depression module of the Patient Health Questionnaire
SBQ-R	Suicide Behaviors Questionnaire-Revised
ERI	Effort–reward imbalance
OR	Odds ratio
CI	Confidence interval
